# Proteomic analysis reveals divergent inflammatory mechanisms of COVID-associated Guillain-Barré syndrome

**DOI:** 10.1007/s00109-026-02698-2

**Published:** 2026-07-04

**Authors:** Can Ulutekin, Amelie Can, Lenka Súkeníková, Luis Querol, Raul Juntas-Morales, Arnau Llauradó, Anelia Dietmann, Tobias Weiss, Daniela Latorre, Burkhard Becher, Florian Ingelfinger, Bettina Schreiner

**Affiliations:** 1 Institute of Experimental Immunology, University of Zurich, Zurich, Switzerland; 2 Department of Neurology, Clinical Neuroscience Center, University Hospital and University of Zurich, Zurich, Switzerland; 3 Institute of Microbiology, ETH Zurich, Zurich, Switzerland; 4 Neuromuscular Diseases Unit, Hospital de la Santa Creu i Sant Pau, IR SANT Pau, Barcelona, Spain; 5 Neuromuscular Diseases Unit, Vall d’Hebron University Hospital, Passeig de la Vall d’Hebron, Barcelona, Spain; 6 Department of Neurology, Inselspital, Bern University Hospital and University of Bern, Bern, Switzerland; 7 Bezirkskrankenhaus Kufstein, Kufstein, Austria; 8 Division of Neuroscience, Department of Neurology, Institute of Experimental Neurology (INSPE), San Raffaele Scientific Institute, Milan, Italy; 9 Department of Medicine I, Medical Center - University of Freiburg, Faculty of Medicine, University of Freiburg, Freiburg, Germany

**Keywords:** SARS-CoV-2, COVID-19, Guillain-Barré syndrome (GBS), Inflammation-related protein biomarkers

## Abstract

Guillain-Barré syndrome (GBS) is an acute immune-mediated neuropathy triggered by infections, with poorly understood pathophysiological diversity. COVID-19-associated GBS (COVID-GBS) is a rare but severe post-infectious condition, and its immune mechanisms remain unclear. We profiled immune mediators in cerebrospinal fluid (CSF) and serum from COVID-GBS patients, comparing them to non-COVID GBS (Control-GBS), COVID-19 patients without neurological complications (COVID-no-GBS) and non-inflammatory neuropathy controls (Neuropathy-no-GBS). To gain mechanistic insights, we integrated publicly available single-nucleus transcriptomic data from sural nerve biopsies of neuropathy patients. IL-8 was confirmed as a key cytokine in GBS. Analysis of publicly available single-nucleus transcriptomic data from non-GBS sural nerve biopsies suggested myeloid cells as potential sources of IL-8, with evidence of autocrine signaling capacity. LIF and CD8A emerged as novel biomarkers, with this transcriptomic analysis indicating that LIF receptor components are expressed on endothelial and stromal cells, suggesting these as potential cellular targets. COVID-GBS patients exhibited unique CSF alterations and distinct serum profiles marked by altered NK cell activity, cytotoxic T-cell responses, and myeloid differentiation. Moreover, associations between inflammatory, extracellular matrix, and regulatory markers with clinical disability differed between COVID-GBS and Control-GBS, pointing to divergent immune mechanisms. Our findings suggest that GBS involves myeloid-driven cytokine responses and local LIF signaling. Analysis of publicly available transcriptomic data from non-GBS sural nerve biopsies suggests potential cellular sources and targets, though validation in GBS-affected tissue is needed. COVID-GBS features a distinct immune signature involving localized and systemic inflammation. These insights deepen our understanding of GBS pathogenesis and nominate candidate biomarkers for further validation and potential therapeutic targeting.

**Supplementary Information:**

The online version contains supplementary material available at 10.1007/s00109-026-02698-2.

## Key messages

CSF proteomics confirm elevated IL-8 and newly identify increased leukemia inhibitory factor (LIF) in Guillain-Barré Syndrome (GBS).

Integration of public transcriptomic nerve biopsy data sets suggests myeloid cells as source of IL-8 and maps LIF receptor (LIFR) to nerve endothelial cells.

COVID-associated GBS has a distinct immune profile compared to pre-pandemic GBS.

Serum proteins OPG and MMP-10 correlate with clinical disability in COVID-associated GBS.

**Supplementary Information:**

The online version contains supplementary material available at 10.1007/s00109-026-02698-2.

## Introduction

GBS is considered a post-infectious disease that affects the peripheral nerve roots and nerves and causes muscle weakness (reviewed in [1]). Preceding infections, reported by approximately two-thirds of GBS patients and most often enteritis or acute respiratory infections, have been associated with specific clinical features and disease severities [2]. Infections that have been linked to GBS in case-control studies include *Campylobacter jejuni*, *Mycoplasma pneumoniae*, cytomegalovirus (CMV), Epstein-Barr virus (EBV), and Zika virus [3, 4]. During the COVID-19 pandemic reports of rare GBS after severe acute respiratory syndrome coronavirus 2 (SARS-CoV2) infections have emerged [5–7]. GBS variants, facial palsy and demyelination appear to be common features [8]. While some case-control studies suggest that SARS-CoV2 infection is associated with an increased risk of GBS [9, 10], other large epidemiologic studies, including a 2021 UK cohort [11] and a 2022 retrospective analysis [12], found no significant association [8]. Thus, although COVID-GBS has been observed, strong evidence of a causal link is lacking, and the underlying pathophysiologic mechanisms remain unclear. Unlike C. jejuni-related GBS, where ganglioside autoantibodies and molecular mimicry are common, in reports of patients with COVID-GBS, anti-ganglioside autoantibodies are mostly absent [8, 13]. This supports the notion that molecular mimicry between Sars-CoV2 and other autoantigens, for example human heat shock proteins [14], or additional immune mediated mechanisms may underlie peripheral nerve injury. Despite COVID-GBS clinical features being similar to classical “post-viral” GBS, a recent study by Súkeníková and colleagues [15] indicated that only a fraction of SARS-CoV2 associated GBS patients displayed an autoreactive T cell response against peripheral nerve myelin antigens. The authors describe a high and sustained background proliferation of T cells from “post-COVID-19” GBS patients and suggest that bystander mechanisms driven by excessive inflammation and soluble mediators may also contribute to disease immunopathology [16].

Due to the rarity of the disease, no available literature has comprehensively characterized the SARS-CoV2-induced inflammatory response in a larger cohort of GBS patients. To better understand COVID-19-associated local and systemic immune changes accompanying GBS, we analyzed cerebrospinal fluid (CSF) and serum collected from patients with COVID-GBS and control groups using a targeted proteomic analysis platform. Our findings indicated that COVID-GBS displayed divergent inflammatory mechanisms, with distinct immune signatures in CSF and serum, unique biomarker profiles, and differential associations with clinical severity compared to non-COVID / Control-GBS patients in a European cohort.

## Methods

### Study design and participants

The study included 57 patients recruited from 4 sites in Switzerland (University Hospital Zurich and Bern), and Spain (Hospital de la Santa Creu i Sant Pau-Barcelona and Vall d`Hebron Barcelona Hospital). COVID-19-related GBS patients with acute neurological phase in April 2020 to February 2022 (3 sites: Zurich, Bern, Vall d`Hebron) and pre-pandemic GBS patients in April 2017 to February 2020 (Zurich) were included in the study. Of note, the first COVID-19 case in Switzerland was confirmed on 25th of February 2020 in the Southern canton Ticino bordering Italy [17]. For this reason, we have chosen this date as the cut-off point to define “pre-pandemic” GBS for Swiss patients. The interval between SARS-CoV-2 infection (or “preceding symptoms” of infection) and GBS onset was recorded, with a mean of 25 days (range 10–50) in the COVID-GBS cohort (Table S1). We also included 3 presumably non-COVID-19-related Control-GBS patients (no typical preceding symptoms, negative SARS-CoV2 PCRs in all 3 patients) during the pandemic with acute GBS manifestations in 2022 (Bern) to have enough samples for analysis. More details about the recruitment of GBS and control patients and samples are described in Figure S1 and Table S1-3. PEX and IVIG treatment dates were recorded for all patients alongside lumbar puncture and serum collection (Table S4). Of 29 patients, 20 samples were collected either prior to IVIG or from patients who did not receive IVIG; one COVID-GBS patient had samples collected immediately after 1–2 PEX sessions and another COVID-GBS patient had samples collected 18 months after their PEX session. Analysis of the first principal components indicated that IVIG could have an impact on protein profiles in the CSF (opposed to serum) but most importantly did not act as confounding variable for the dissection of COVID-GBS patients compared to Control-GBS (Figure S3). The Neuropathy-no-GBS control group comprised patients with clinically and electrophysiologically confirmed non-inflammatory neuropathies, with genetic confirmation where applicable; individuals with a history of GBS or other acute autoimmune neuropathies were excluded. Specific diagnoses are summarized in Table S3.

The COVID-no-GBS control group comprised 10 COVID-19 patients without GBS who underwent lumbar puncture for neurological manifestations unrelated to GBS during the pandemic. The mean age was 51 years (range 29–75), with 60% male (Table S1). All patients had confirmed SARS-CoV-2 infection by PCR and reported typical COVID-19 symptoms including fever, respiratory symptoms, fatigue, anosmia, headache, and myalgia; one patient also experienced cough and diarrhea. The time interval between COVID-19 and lumbar puncture was 58 days on average. Indications for lumbar puncture included fatigue (*n* = 3), cerebrovascular stroke (*n* = 3), headache (*n* = 1), epileptic seizure (*n* = 1), and functional disorder (*n* = 1) (Table S3). Five patients (50%) were hospitalized for neurological diagnostic work-up, but none experienced severe COVID-19 pneumonia or complications such as ICU admission, ARDS, or mechanical ventilation (Table S1). Two patients had oligoclonal bands in both serum and CSF, indicating a systemic immune response (Table S3). CSF analysis showed a mean cell count of 2.5 cells/µL (range 0–10) and mean protein of 0.4 g/L (range 0.3–0.6), with elevated protein (> 0.45 g/L) in 3 patients (30%) (Table S2).

Diagnosis was done based on the criteria for GBS of the National Institute of Neurological Disorders and Stroke (NINDS) [18]. Clinical data on demographics, symptoms of preceding infections and GBS, including routine CSF parameters, nerve conduction studies and treatments were extracted retrospectively from clinical patient records. Disease severity was graded by the local investigator using the GBS disability six-point scale as follows: 0 = healthy, 1 = minor symptoms but capable of running, 2 = able to walk 10 m without assistance but unable to run, 3 = able to walk 10 m with help, 4 = bedridden or chair bound, 5 = requiring assisted ventilation for at least part of the day, 6 = dead [19]. The Medical Research Council (MRC) Sum Score measures muscle strength and ranges from 0 (complete paralysis) to 60 (normal strength) [20]. The electrophysiological classification was done according to Hadden et al. [21] into 5 groups: demyelinating, axonal, equivocal, inexcitable and normal.

All patients with COVID-GBS were confirmed COVID-19 cases meeting the laboratory criteria based on the European Centre for Disease Prevention and Control (ECDC) and World Health Organization (WHO) laboratory recommendations [22, 23].

### Anti-ganglioside antibody screening in serum

Screening for anti-ganglioside antibodies in serum was carried out at hospital sites in Switzerland and Spain as part of routine clinical diagnostics. In Zurich and Bern, the GanglioCombi^®^ MAG ELISA (Bühlmann Laboratories; detecting anti-GD1a, GD1b, GM1, GT1a, and GQ1b) was performed according to the manufacturer’s instructions. At the Hospital de la Santa Creu i Sant Pau (Barcelona), patients’ sera were screened for anti-ganglioside antibodies using a previously validated ELISA protocol [24] as a general detection method, and thin-layer chromatography [25] used for confirmatory analyses. Anti-ganglioside antibodies were considered positive at a 1:1000 titer. For patients tested at Vall d’Hebron University Hospital, anti-ganglioside antibody testing was performed using a panel-based line blot assay (EUROIMMUN, Germany), detecting both IgG and IgM antibodies against GM1, GM2, GM3, GD1a, GD1b, GQ1b, and GT1b.

### Proteomic analysis

A total of 92 analytes, including cytokines, chemokines and soluble membrane proteins, were measured in 58 serum and 38 CSF supernatant samples using the Olink^®^ 96 target inflammation panel. The Olink^®^ platform is based on oligonucleotide antibody-pairs that contain unique DNA sequences. Matched DNA reporter pairs hybridize to produce an amplicon for real-time qPCR. The measurements were performed by the Olink^®^ Analysis Service at the Swiss Institute of Allergy and Asthma Research (SIAF, Davos, Switzerland). The Olink^®^ Inflammation panel reports the analyte S100A12; we use the functional designation EN-RAGE (Extracellular Newly identified RAGE-binding protein) throughout the manuscript, with S100A12 noted parenthetically at first mention in each major section. Olink^®^ data for both CSF and serum samples was acquired in two separate runs on different dates, with some samples overlapping between runs. These overlapping samples were used to guide integration of the two datasets via the “olink_normalization” function in the OlinkAnalyze R package. Samples failing quality control (QC) were excluded from further analysis. CSF exclusions included samples from two COVID-no-GBS patients, one Control-GBS patient, and one Neuropathy-no-GBS patients. Serum exclusions comprised samples from three COVID-GBS patients, one COVID-no-GBS patient, one Control-GBS patient, and four Neuropathy-no-GBS patients. The number of CSF and serum samples analyzed for each patient group after QC is summarized in Table S5. The remaining data was standardized by applying z-score transformation (using the base R “scale” function). Finally, for each analysis, assays were excluded if more than 30% of their measurements fell below the limit of detection. To evaluate potential batch effects related to cohort origin, we performed principal component analysis (PCA). Principal component analysis (PC1 and PC2) of scaled protein abundance in both CSF and serum demonstrated clear separation aligned with clinical patient stratification, confirming that cohort origin did not obscure key disease-related patterns (Figure S2A). We sought to correlate principal components with clinical parameters to determine their impact on the patients’ proteomic landscape. Regarding time from GBS onset to sampling, ICU admission, and mechanical ventilation, we observed no clear pattern indicating that these factors influenced the proteomic profile of the combined GBS cohorts (Figure S2B). However, hospitalization and complications related to COVID-19 did appear to impact the proteomic landscape of COVID-GBS and COVID-no-GBS patients (Figure S2C), suggesting that COVID-19 severity and related factors could affect proteomic differences.

### Algorithm-guided and statistical analyses

All statistical analyses were done in R. Comparisons between groups were done using the non-parametric unpaired Wilcoxon rank-sum test. Corrections for multiple hypothesis testing were done using the Benjamini-Hochberg method and were performed for each of the soluble protein classifications (Signaling Molecules, Cytokines, Chemokines, Growth Factors and Others). Clinical correlations were done using Spearman’s method. Statistical methods are indicated in figure legends. Principal component analysis (PCA) was done using the “prcomp” function from the stats package.

Publicly available single-nucleus RNA sequencing data from Heming et al. [26], derived from sural nerve biopsies of patients with polyneuropathy and controls, were used for gene expression analysis and visualization. The R package Seurat was used to generate UMAP and dot plots using merged annotations based on the original clustering provided by Heming et al., consolidated into broader cell populations. The data was already scaled and normalized by the authors.

To dissect COVID-GBS signatures into COVID-19-like, GBS-like or unique to COVID-GBS we employed the Anderson-Darling K-sample test, from the R package “kSamples”. This is a non-parametric test used to determine whether independent samples come from the same underlying distribution. The lower the standardized test statistic implies that the data from the two groups share the same underlying distribution whereas higher values imply a different underlying distribution. When modeling distinguishing biomarkers between patient groups, candidate biomarkers were first screened with the Boruta function from the R package “Boruta”. Confirmed proteins were forwarded to a random-forest classifier (R package “randomForest”). Because of the small cohort size, a leave-one-out cross-validation (LOOCV) loop was used. The AUROC for this model was then calculated with the R package “pROC”.

## Results

### Proteomic analysis reveals interleukin (IL)-8 and LIF as CSF biomarkers for GBS

**Fig. 1:**
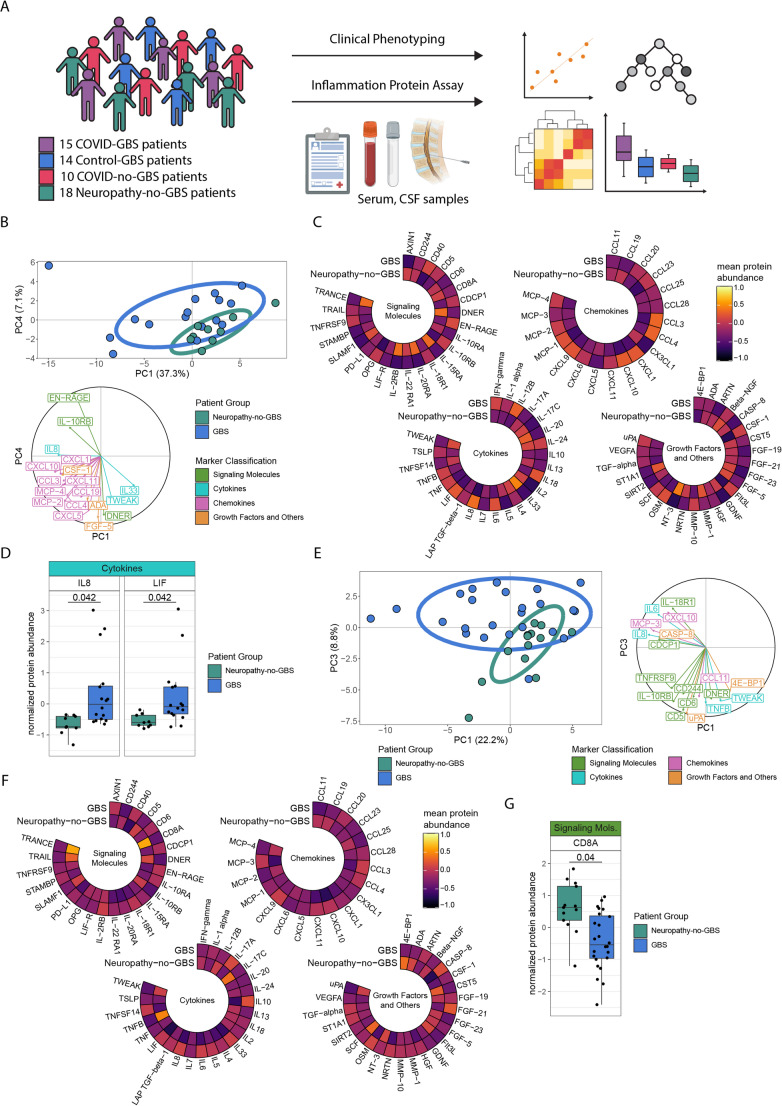
IL-8, LIF, and soluble CD8A mark distinct immune alterations in GBS CSF and serum. **A** Schematic overview depicting patient groups with clinical data used for phenotyping, and serum and CSF samples analyzed by inflammation protein assay. Results were integrated and analyzed using advanced bioinformatics techniques to uncover key immune aspects of peripheral nerve inflammation. The patients in the pre-/pandemic GBS group had no prior COVID-19. (B-D) CSF samples of 16 GBS patients compared to 9 Neuropathy-no-GBS patients. **B** PCA based on scaled expression of markers, where each dot denotes one patient. Vectors of markers in the circle denote top contributors to PC1 and PC4. **C** Heatmaps illustrating mean scaled marker expressions comparing GBS and Neuropathy-no-GBS patients. Markers are assigned to mutually exclusive categories for ease of representation. **D** Boxplots comparing scaled expression of IL-8 and LIF between GBS and Neuropathy-no-GBS patients. Unpaired Wilcoxon rank-sum test and Benjamini-Hochberg correction were applied. **E**-**G** Serum samples of 24 GBS patients compared to 13 Neuropathy-no-GBS patients. **E** PCA based on scaled expression of markers, where each dot denotes one patient. Vectors of markers in the circle denote top contributors to PC1 and PC4. **F** Heatmaps illustrating mean scaled marker expressions comparing GBS and Neuropathy-no-GBS patients. Markers are assigned to mutually exclusive categories for ease of representation. **G** Boxplot comparing scaled expression of CD8A between GBS and Neuropathy-no-GBS patients. Unpaired Wilcoxon rank-sum test and Benjamini-Hochberg correction were applied

To characterize immune responses in COVID-GBS, we performed multiplexed proteomic profiling of CSF and serum using the Olink^®^ Target 96 Inflammation panel. We analyzed samples from COVID-GBS and Control-GBS patients at the time of diagnosis, as well as two control groups: COVID-19 patients without neurological complications (COVID-no-GBS) and patients with neuropathies unrelated to COVID-19 or GBS (Neuropathy-no-GBS) (Fig. 1A). Clinical, electrophysiological, and outcome data are provided in Supplementary Tables S1-S3 and Figure S1. We could not detect direct viral neuroinvasion at the timepoint of CSF sampling to explain peripheral neurological dysfunction and a persistent hyperinflammatory state in our COVID-GBS patients (Table S2).

To extract a protein signature associated with GBS irrespective of their viral association, we compared the protein abundance in the CSF of all GBS patients with those of Neuropathy-no-GBS patients. Principal component analysis (PCA) revealed a broad variation within GBS patients compared to the Neuropathy-no-GBS patients indicating that the underlying molecular features might be more heterogenous than in the control group (Fig. 1B). Moreover, principal components (PC) 1 and 4 separated the CSF protein profiles of GBS and Neuropathy-no-GBS patients (Fig. 1B). Next, soluble proteins were grouped into four mutually exclusive categories: “signaling molecules”, “cytokines”, “chemokines” and “growth factors and others” and their abundance was compared between GBS and control patients (Fig. 1C). We observed that the cytokines IL-8 and leukemia inhibitory factor (LIF) were significantly increased in GBS patients compared to Neuropathy-no-GBS patients at the time of the initial diagnostic workup (Fig. 1D). IL-8 is a proinflammatory chemokine that recruits neutrophils and other immune cells to sites of inflammation and promotes vascular permeability [27]. Increased abundance of IL-8 in the CSF of GBS and chronic inflammatory demyelinating polyradiculopathy (CIDP) patients is well-supported in the literature [28, 29]. LIF, a cytokine of the IL-6 family, has been shown to have protective functions in inflammation and Schwann cell survival [30, 31]. Therefore, elevated LIF in the CSF of GBS patients could reflect a regenerative response to immune-mediated nerve damage. Next, we interrogated to what extent these CSF biomarkers were also elevated in the serum of GBS patients. IL-8 and LIF were not significantly elevated in serum, indicating their role may be restricted to the peripheral nerve compartment (Figure S4A).

Taken together, our findings identified a unique immune signature comprising increased abundance of IL-8 and LIF in the CSF of patients with GBS compared to Neuropathy-no-GBS patients.

### GBS patients display reduced serum levels of soluble CD8A

To explore accessible serum biomarkers for GBS, we compared serum protein profiles of GBS and Neuropathy-no-GBS patients. PCA revealed separation between GBS and control patients along PC1 and PC3, driven primarily by surface signaling proteins (Fig. 1E). Among cytokines, chemokines, signaling proteins, and growth factors, CD8A was significantly reduced in the serum of GBS patients (Fig. 1F-G). This reduction may reflect transient lymphopenia, commonly observed in acute (viral) infections [32]. SARS-CoV-2 infection can induce prolonged and severe lymphopenia marked by decreased T-cell counts, with several mechanisms proposed [33]. Thus, lower CD8A levels may indicate a systemic immune response to prior viral infection rather than a direct consequence of neuroinflammation in GBS. The reduction in CD8A levels was limited to the serum; CSF CD8A levels showed no significant difference between GBS and Neuropathy-no-GBS patients (Figure S4B).

In conclusion, our serum protein analysis revealed a reduction in soluble CD8A in GBS patients.

### Cell-type specific expression of IL-8 and LIF signaling components in human peripheral nerve tissue

**Fig. 2:**
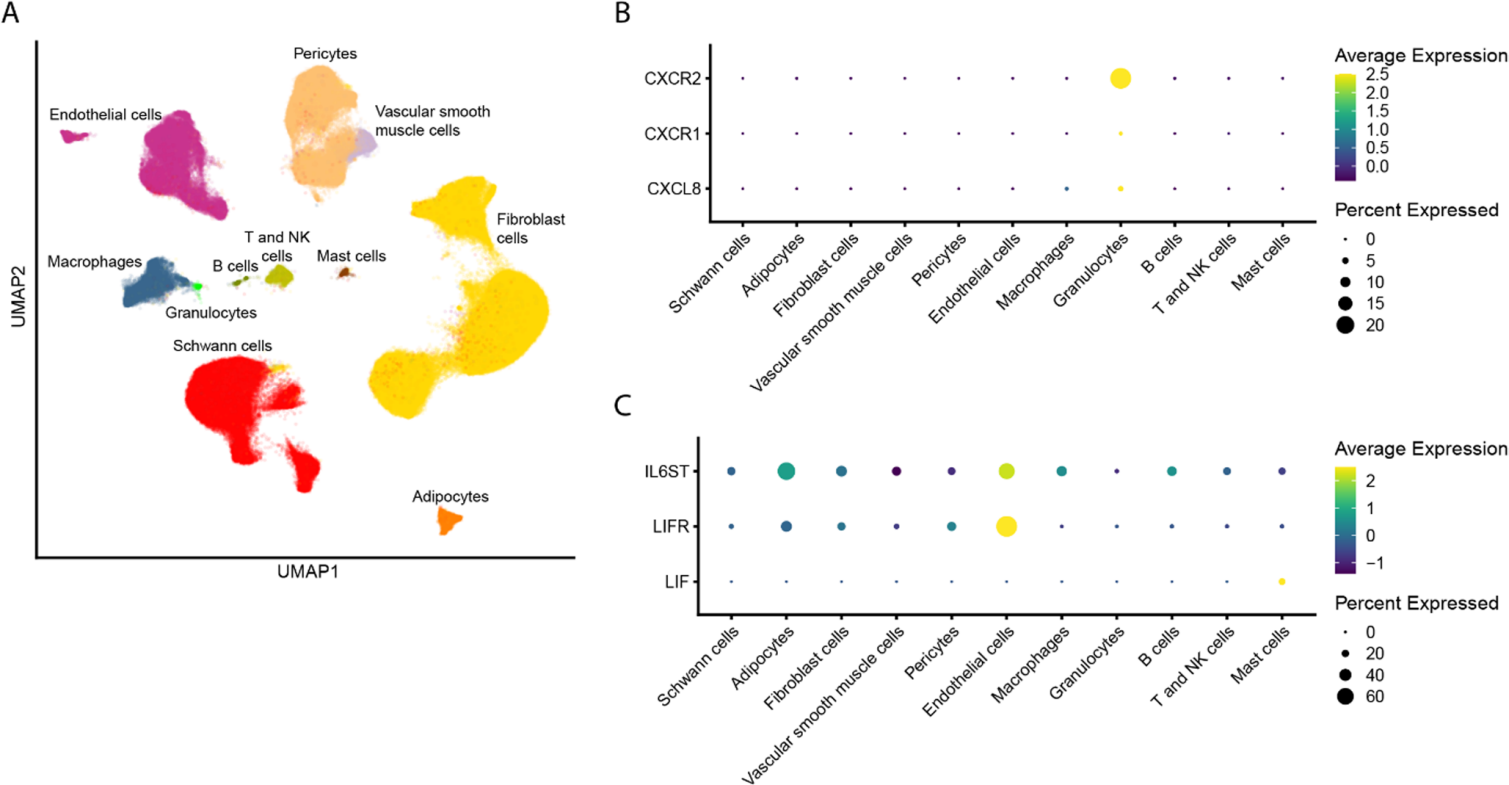
CXCL8 (IL-8), LIF, and their receptors’ expression across cell types in human peripheral nerves. **A**-**C** Single-nucleus RNA sequencing data from Heming et al. [26] derived from sural nerve biopsies of patients with polyneuropathy and controls. **A** UMAP plot showing the cellular composition of peripheral nerves. Cell clusters reflect merged annotations based on Heming et al., consolidated into broader cell populations. **B** Dot plot illustrating gene expression of IL-8 (*CXCL8*) along with its receptors *CXCR1* and *CXCR2* across major identified clusters. Dot size indicates the proportion of nuclei expressing each gene within a cluster; color intensity reflects the average expression level. **C** Dot plot illustrating gene expression of *LIF* along with its receptors *LIFR* and *IL6ST* across major identified clusters. Dot size indicates the proportion of nuclei expressing each gene within a cluster; color intensity reflects the average expression level

To explore potential cellular sources and targets of IL-8 and LIF signaling within peripheral nerve tissue, we examined their expression patterns using publicly available transcriptomic data. Because nerve biopsies are not routinely performed in GBS patients, we leveraged publicly available single-nucleus transcriptomic data from Heming et al. [26], derived from sural nerve biopsies of patients with polyneuropathies and non-neuropathic controls. This independent dataset enabled us to interrogate which peripheral nerve-resident cell types may contribute to or respond to IL-8 and LIF signaling. Consistent with known roles of IL-8, this analysis revealed *CXCL8* expression, the transcript encoding for IL-8, predominantly in myeloid populations, with the highest levels and proportions of *CXCL8*⁺ cells found in granulocytes (Fig. 2A and B). Conversely, the receptors for IL-8, *CXCR1* and *CXCR2*, were primarily expressed by granulocytes, suggesting a local myeloid environment capable of both producing and sensing IL-8 (Fig. 2B). These observations align with prior reports of neutrophil-intrinsic IL-8 dysregulation contributing to inflammatory processes [34].

In contrast, *LIF* demonstrated highest average expression observed in a small subset of mast cells (Fig. 2C). Its receptor, *LIFR*, was more broadly expressed across several non-immune resident cell types, most notably endothelial cells (Fig. 2C). Additional expressions of *LIFR* were observed in various stromal cell types, suggesting that LIF signaling may primarily act on vascular and stromal components of the nerve. *IL6ST* (also known as gp130), a shared signal-transducing co-receptor required for downstream signaling of LIF and other IL-6 family cytokines, was also highly expressed in endothelial cells, perineurial fibroblasts, and adipocytes - overlapping with *LIFR*-expressing populations and supporting their capacity to respond to LIF. In contrast, *IL6ST* expression was also found in subsets of immune cells, including macrophages and B cells, which largely lacked *LIFR*, suggesting possible responsiveness to other IL-6 family cytokines rather than to LIF specifically (Fig. 2C). Of note, differential comparison of non-inflammatory and inflammatory neuropathies, including CIDP, vasculitic neuropathy, and other inflammatory forms, revealed cell-type-specific regulation of *LIFR* and *IL6ST* during inflammation (Figure S5A and B).

While these data derive from non-GBS neuropathy cases and sural nerve tissue that may not reflect the primary pathological sites in GBS (nerve roots, proximal nerves where ‘sural sparing’ is a recognized feature), they provide preliminary, hypothesis-generating insight into peripheral nerve-residing cell populations potentially involved in IL-8 and LIF signaling in GBS.

### SARS-CoV-2-linked GBS patients have a unique serum protein profile versus COVID-19 or GBS controls

**Fig. 3:**
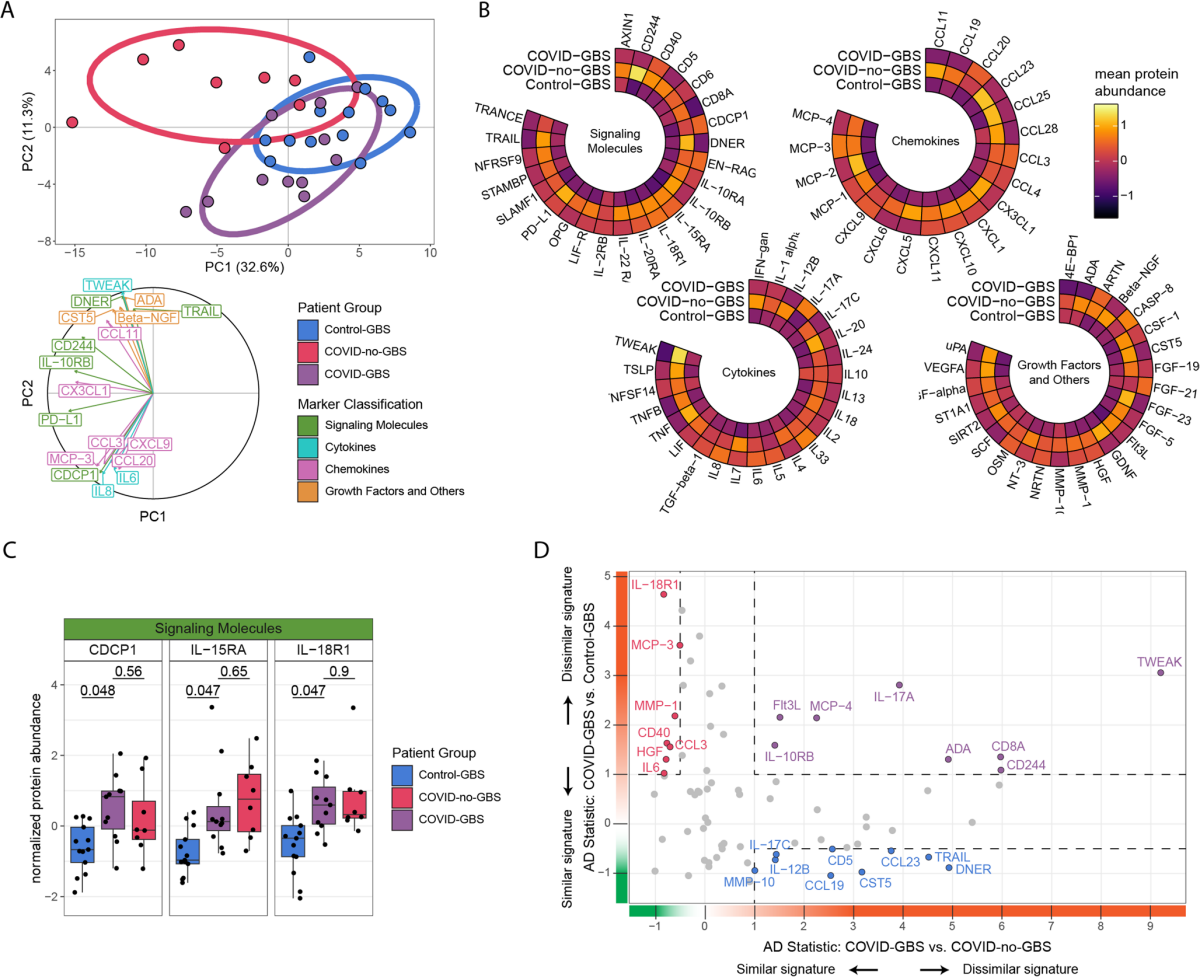
Serum proteomics reveal divergent immune landscapes among COVID-GBS, COVID-no-GBS, and Control-GBS patients. **A**-**C** Serum samples of 11 COVID-GBS patients compared to 8 COVID-no-GBS patients and 13 Control-GBS patients. **A** PCA based on scaled expression of markers, where each dot denotes one patient. Vectors of markers in the circle denote top contributors to PC1 and PC2. **B** Heatmaps illustrating mean scaled marker expressions comparing COVID-GBS patients to COVID-no-GBS and Control-GBS patients. Markers are assigned to mutually exclusive categories for ease of representation. **C** Boxplots comparing scaled expressions of CDCP1, IL-15RA and IL-18R1 in COVID-GBS patients to COVID-no-GBS and Control-GBS patients. Unpaired Wilcoxon rank-sum test and Benjamini-Hochberg correction was applied. **D** Serum samples of 11 COVID-GBS, 8 COVID-no-GBS and 13 Control-GBS patients. Scatter plot showing Anderson-Darling (AD) statistics comparing protein expression distributions. X-axis represents the AD statistic for COVID-GBS versus Control-GBS; Y-axis represents the AD statistic for COVID-GBS versus COVID-no-GBS. Lower (more negative) values indicate greater similarity in distribution between groups, while higher (more positive) values indicate distinct distributions. Cut-offs: -0.5 for similarity, 1.0 for dissimilarity

We next investigated whether COVID-GBS may exhibit molecular features that differ from those observed in Control-GBS and COVID-no-GBS patients (Fig. 3).

Similar to the broader GBS group, COVID-GBS patients showed marked variability in CSF protein profiles (Figure S6A-B). However, the limited availability of CSF samples from COVID-GBS patients (*n* = 4), attributable to reduced lumbar punctures during the pandemic and post-acute viral illness, precluded robust CSF-based analyses; therefore, CSF findings are presented only as preliminary, hypothesis-generating assessments in the Supplement (Figure S6A-B, Figures S7A-B).

Serum proteomics of COVID-GBS patients revealed a distinctive profile combining features of both COVID-19 and GBS pathophysiology (Fig. 3A and B). Discriminatory proteins between Control-GBS and COVID-GBS were enriched among signaling molecules (Fig. 3C). Notably, signaling proteins including the T cell trafficking factor CDCP1 and cytokine receptors IL-15RA and IL-18R1 were elevated in both COVID-GBS and COVID-no-GBS groups but were markedly lower in Control-GBS patients. Increased soluble IL-15RA and IL-18R1 may reflect compensatory immune regulation dampening NK and CD8⁺ T cell activation in COVID-19, absent in Control-GBS, indicating mechanistic differences between COVID-GBS and Control-GBS.

Furthermore, we used the Anderson-Darling k-sample test to identify which protein features in COVID-GBS overlap with or differ from those in COVID-no-GBS and Control-GBS groups. This test assesses whether protein levels are similarly distributed across groups. Serum analysis revealed that COVID-GBS shares some features with each group but remains distinct overall (Fig. 3D and S7C).

### IL-7 and EN-RAGE distinguish COVID-GBS from Control-GBS

**Fig. 4:**
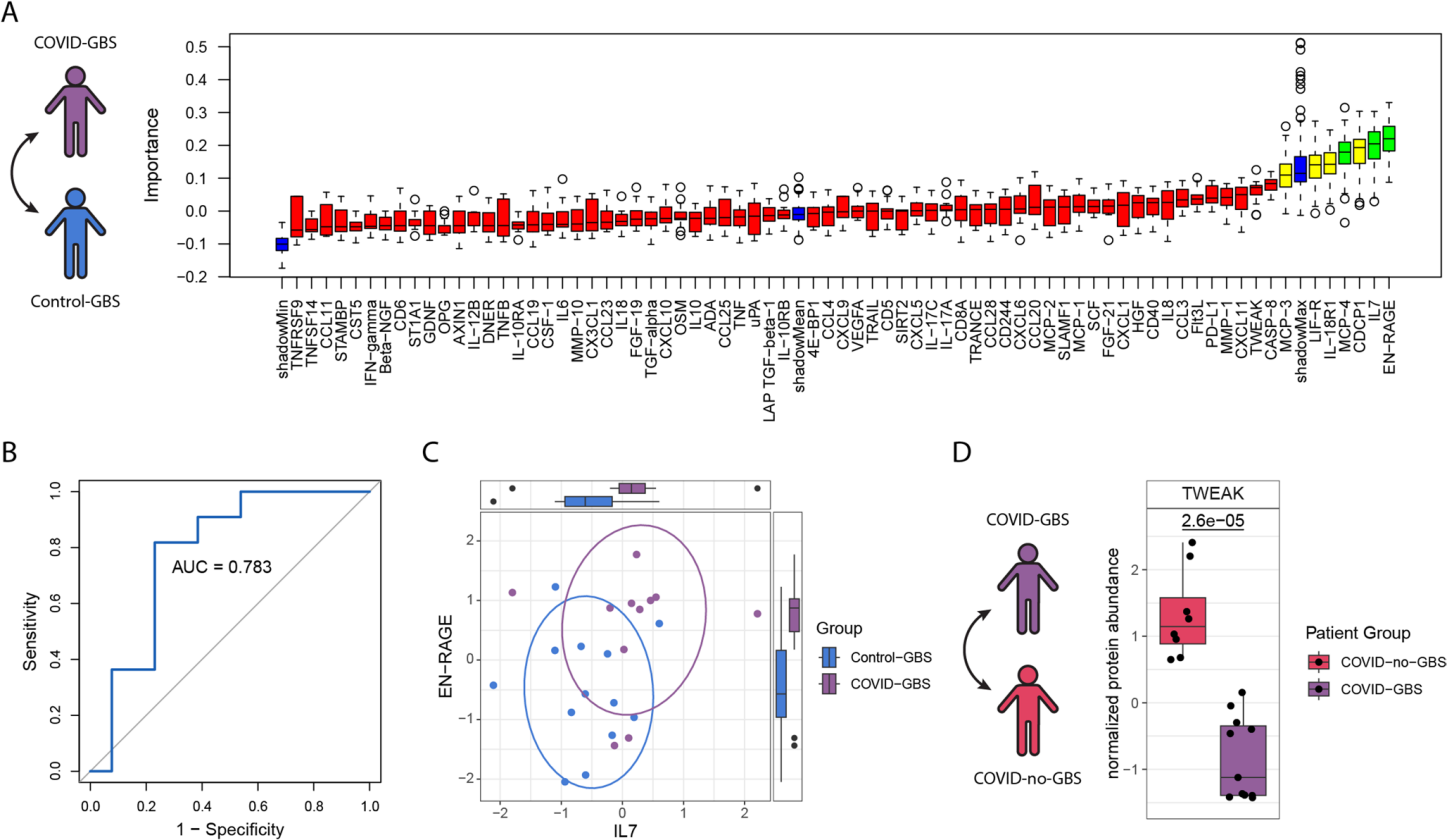
EN-RAGE and IL-7 discriminate COVID-GBS from Control-GBS. **A** Boxplots from the Boruta feature selection algorithm, used to classify COVID-GBS patients from Control-GBS patients. Features useful for classification are shown in green, rejected features in red, and uncertain features in yellow. **B** ROC curve for random-forest model using features IL7, EN-RAGE for discriminating COVID-GBS patients from Control-GBS patients. Leave-One-Out Cross-Validation (LOOCV) method was used to validate the model. **C** Dot plot showing scaled expression of IL7 and EN-RAGE in COVID-GBS and Control-GBS patients. **D** Boxplot comparing scaled expression of TWEAK in COVID-GBS patients to COVID-no-GBS patients. Unpaired Wilcoxon Rank sum test was used

To identify serum proteins that best differentiate COVID-GBS patients from Control-GBS patients, we trained a random forest classifier. IL-7 and EN-RAGE (S100A12) emerged as the top features distinguishing COVID-GBS from Control-GBS (Fig. 4A). Using these markers, a Leave-One-Out Cross-Validation (LOOCV) showed good classification performance (AUC: 0.783) (Fig. 4B). COVID-GBS patients had higher serum levels of IL-7 and EN-RAGE compared to controls (Fig. 4C).

Next, we interrogated serum proteins to distinguish COVID-GBS patients from unrelated COVID patients (COVID-no-GBS). TWEAK protein levels in the serum of COVID-GBS patients was lower compared to COVID-no-GBS controls and facilitated a clear separation boundary (Fig. 4D). These findings highlight a unique serum protein signature for COVID-GBS, warranting validation in larger cohorts for clinical use.

### GBS disability links differently to OPG, MMP-10, IL-10Ra in patients with COVID

**Fig. 5:**
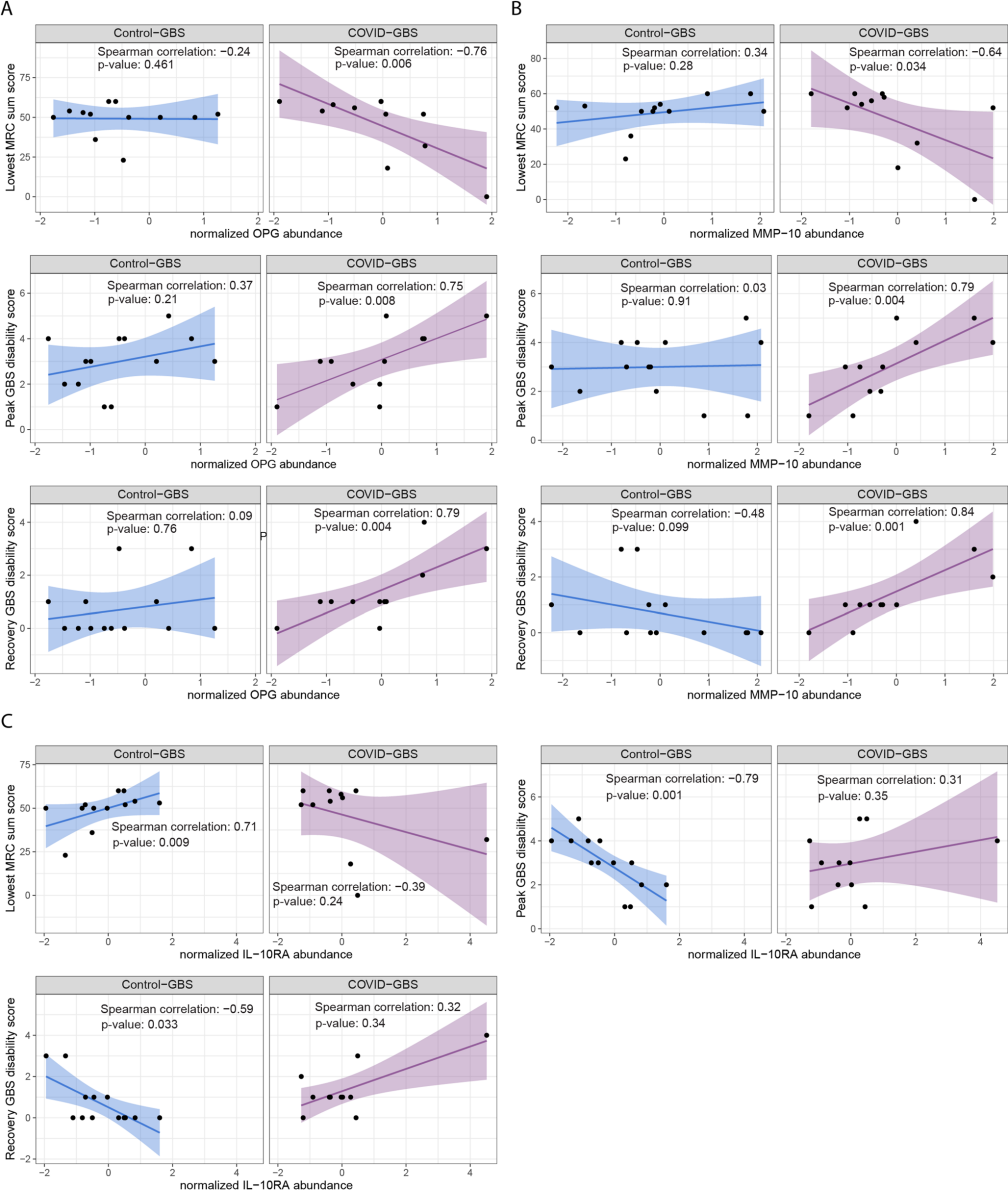
Differential associations of OPG, MMP-10, and IL-10RA levels with GBS disability distinguish COVID-GBS from Control-GBS. **A** Scatter plots displaying scaled serum OPG expression (x-axis) in relation to three clinical outcome measures: lowest MRC sum score (top panels), peak GBS disability score (middle panels) and recovery GBS disability score (bottom panels), shown for Control-GBS (left panels) and COVID-GBS (right panels). Each dot represents an individual patient. Trendlines depict the direction of the association, and Spearman correlation coefficients with corresponding p-values are provided within each panel. **B** Scatter plots displaying scaled serum MMP-10 expression (x-axis) in relation to three clinical outcome measures: lowest MRC sum score (top panels), peak GBS disability score (middle panels) and recovery GBS disability score (bottom panels), shown for Control-GBS (left panels) and COVID-GBS (right panels). Each dot represents an individual patient. Trendlines depict the direction of the association, and Spearman correlation coefficients with corresponding p-values are provided within each panel. **C** Scatter plots displaying scaled serum IL-10RA expression (x-axis) in relation to three clinical outcome measures: lowest MRC sum score (top-left panels), peak GBS disability score (top-right panels) and recovery GBS disability score (bottom panels), shown for Control-GBS (left panels) and COVID-GBS (right panels). Each dot represents an individual patient. Trendlines depict the direction of the association, and Spearman correlation coefficients with corresponding p-values are provided within each panel

To explore the relationship between circulating biomarkers and clinical severity in GBS, we next investigated whether serum protein levels (measured at the time of diagnostic work-up) were associated with key outcome measures. We focused on three clinical parameters: peak GBS disability score, recovery GBS disability score and lowest Medical Research Council (MRC) sum score. Using Spearman correlation analysis, we assessed the direction and strength of associations between selected biomarkers and these clinical outcomes, separately in COVID-GBS and Control-GBS patient groups. Serum osteoprotegerin (OPG) and matrix metalloproteinase-10 (MMP-10) levels correlated positively with both peak and recovery disability in COVID-GBS patients - but not in Control-GBS patients - and negatively with the lowest MRC sum score, indicating a more severe clinical presentation (Fig. 5A and B). OPG, also known as tumour necrosis factor receptor superfamily member 11B (TNFRSF11B), functions as a decoy receptor for RANKL, preventing the RANK–RANKL interaction that typically stimulates osteoclastogenesis [35]. MMPs, including MMP-10, degrade extracellular matrix components, disrupt the blood–nerve barrier, facilitate immune cell infiltration, and contribute to inflammatory demyelinating diseases. In contrast, soluble IL-10 receptor alpha (IL-10RA) levels were negatively correlated with both peak and recovery disability in Control-GBS patients, but not in COVID-GBS, and were positively correlated with the lowest MRC sum score, suggesting that higher sIL-10RA levels are associated with less severe disease (Fig. 5C).

Together, these results underscore the complex interplay of inflammatory mediators and their potential role in the pathophysiology of GBS, with distinct mechanisms at play in COVID-no-GBS and Control-GBS.

## Discussion

We first assessed whether GBS, regardless of its association with SARS-CoV-2, showed a distinct proteomic signature compared to Neuropathy-no-GBS. Elevated IL-8 in CSF was a key feature, in line with existing literature [28, 29, 36]. IL-8 (CXCL8) can act as a strong chemoattractant through CXCR1 and CXCR2, promote vascular permeability, and facilitate immune cell infiltration into nerve roots and peripheral nerves [37]. Beyond immune cell trafficking, CXCR2 is also expressed in peripheral sensory neurons (and the spinal cord), where it may contribute to inflammatory pain [38]. Analysis of publicly available single-nuclei transcriptomic data from sural nerve biopsies of non-GBS polyneuropathy patients suggested IL-8 expression primarily in myeloid cells, while CXCR1 and CXCR2 were largely restricted to granulocytes - suggesting potential IL-8-mediated signaling within the myeloid compartment of the nerve. Although Kmezic et al. found a correlation between CSF IL-8 and clinical severity in GBS, we did not replicate this (Figure S8), possibly due to disease heterogeneity, or methodological / sampling differences [28]. In addition, LIF was significantly elevated in the CSF of GBS patients compared to Neuropathy-no-GBS, reflecting a potential protective response to nerve injury. LIF receptor (LIF-R), expressed on Schwann cells and peripheral nerves, including nerve roots, may mediate neuroprotective and regenerative effects [39]. Analysis of publicly available transcriptomic data from non-GBS sural nerve biopsies indicated LIFR and its co-receptor IL6ST expression in endothelial and stromal cells, suggesting these cell types as potential targets of LIF signaling, though this requires validation in GBS-affected nerve tissue. Similar LIFR levels in COVID- and Control-GBS suggest this pathway is active regardless of infectious trigger (Figure S6 and S7). Agents targeting IL-8-related pathways-including CXCR1/2 antagonists (e.g., reparixin [40], navarixin [41]) or IL-8-neutralizing antibodies (e.g., HuMax-IL8, BMS-986253 [42]), as well as approaches to enhance LIF signaling (e.g., modulation of soluble LIF receptor activity) - have been explored in other inflammatory or repair contexts and could be considered for future study in GBS. These therapeutic considerations are speculative and intended only to highlight directions for future research.

Next, we focused on COVID-GBS cases to dissect potential virus-specific immune mechanisms and their contribution to nerve inflammation. Due to the limited availability of CSF samples from COVID-GBS patients, we analyzed serum profiles and identified IL-7 as a key differentiator compared to Control-GBS. Elevated IL-7 (a cytokine essential for T-cell survival and homeostasis) may reflect a compensatory response to T-cell depletion [43], consistent with reports of high background proliferation and limited autoreactivity in COVID-GBS [15]. This supports a model of bystander-driven inflammation rather than targeted autoreactivity [43]. Soluble IL-15RA and IL-18R1 were elevated in both COVID groups, potentially limiting cytotoxic T or NK cell overactivation. These findings suggest that dysregulated, non-antigen-specific immune activation may contribute to COVID-GBS pathogenesis. Notably, Kim et al. [44] investigated soluble factors such as IL-15 in acute hepatitis A and showed that they could modulate innate-like, TCR-independent cytotoxicity driven by bystander CD8⁺ T-cell activation - an alternative pathogenic route not reliant on classical autoimmunity [45, 46].

Innate immune dysregulation also emerged as a distinguishing feature. EN-RAGE (S100A12), a myeloid-derived damage-associated molecular pattern protein (DAMP), was a top classifier in distinguishing COVID-GBS from Control-GBS. EN-RAGE accumulates at sites of inflammation and acts as a potent chemoattractant. It is also associated with cortical brain alterations in COVID-19 patients exhibiting neurological symptoms suggestive of immune-mediated effects on nervous tissue [47, 48]. TWEAK, a member of the TNF superfamily, was another discriminative cytokine that distinguished COVID-GBS from COVID-no-GBS. TWEAK binds to its receptor, Fn14 (fibroblast growth factor-inducible 14), expressed on both immune and non-immune cells, activating signaling pathways that - when overactivated, as in COVID-19 - can amplify inflammation, tissue damage, and fibrosis [49, 50]. While elevated TWEAK levels in COVID-no-GBS may reflect broader systemic inflammation, its lower levels in COVID-GBS suggest a more localized immune response targeting peripheral nerves. Together, these data reveal a distinct innate immune profile in COVID-GBS, involving EN-RAGE-driven myeloid activation and more compartmentalized inflammation, separating it from systemic Control-GBS and COVID-no-GBS.

To explore the relationship between inflammation and clinical severity in GBS, we assessed serum biomarkers in relation to functional outcomes. In COVID-GBS patients, elevated levels of OPG and MMP-10 were associated with more severe clinical presentations, including higher GBS disability scores and lower MRC sum scores. OPG (TNFRSF11B), traditionally recognized as a decoy receptor for RANKL in bone remodeling, has emerged as a marker of systemic inflammation in various conditions including cardiovascular disease and vascular calcification [51]. However, the specific mechanistic role of OPG in peripheral nerve inflammation and GBS pathophysiology remains unclear. Several non-mutually exclusive mechanisms may explain the association between elevated OPG and disease severity in COVID-GBS. First, OPG elevation may simply reflect the intensity of systemic inflammatory activation in COVID-19, serving as a surrogate marker rather than a direct pathogenic mediator in peripheral nerve injury. Second, emerging evidence suggests that OPG may have immunomodulatory functions beyond bone metabolism, potentially influencing cytokine networks and immune cell activation, though these mechanisms have not been characterized in the context of peripheral neuropathies. Third, given that OPG is produced by endothelial cells and can be induced by inflammatory cytokines including TNF-α and IL-1β, its elevation may reflect endothelial activation and blood-nerve barrier dysfunction - processes known to facilitate immune cell infiltration in GBS. Importantly, the correlation between OPG levels and clinical severity was observed specifically in COVID-GBS but not in Control-GBS patients, suggesting that this association may be related to COVID-19-specific systemic inflammatory processes rather than a universal feature of GBS pathophysiology. Whether OPG plays a direct pathogenic role in COVID-GBS or serves as a biomarker of disease severity driven by other mechanisms requires further investigation. MMP-10, a matrix-degrading enzyme, likely contributes to blood-nerve barrier disruption and immune cell infiltration, which aligns with its involvement in neuroinflammation and tissue injury in inflammatory neuropathies [52]. Furthermore, Bonetto et al. reported persistently elevated circulating MMP-9 levels in COVID-19 patients with neurological complications, linking matrix remodeling enzymes to blood-brain barrier dysfunction in both acute and chronic phases of SARS-CoV-2 infection [53]. In contrast, soluble IL-10RA levels were inversely correlated with disability in Control-GBS, but not in COVID-GBS, suggesting a protective, anti-inflammatory role in non-COVID GBS. The lack of correlation in COVID-GBS points to potential disruption of IL-10 signaling by SARS-CoV-2, overriding its typical regulatory effect on inflammation [54]. These findings underscore the potential impact of underlying systemic inflammation, blood-nerve barrier integrity, and the functionality of regulatory cytokine pathways on the clinical outcomes of GBS, depending on the infection-associated context.

While our study examines GBS following SARS-CoV-2 infection, we do not make claims about a causal relationship. COVID-associated GBS is used as a descriptive term for temporal association, with some studies suggesting increased risk [9, 55] and others finding no significant link [11, 12]. Our findings focus on inflammatory CSF and serum protein profiles in these patients, providing molecular insights into potential differences from non-COVID GBS without implying causality.

### Limitations of the study

Although the study included multiple centers and control groups, sample sizes were limited; therefore, our findings should be interpreted as exploratory and hypothesis-generating. Larger, prospective studies with functional validation are needed to confirm and extend these findings. Profiling soluble proteins in CSF and serum enables biomarker identification but offers limited mechanistic insight, including cellular origin. Due to lack of nerve tissue from GBS patients, we used publicly available single-nuclei transcriptomic data from non-GBS polyneuropathies to infer potential cellular sources. Importantly, these data were derived from sural nerve biopsies, which do not reflect the primary pathological sites in GBS (nerve roots, proximal nerves) where ‘sural sparing’ is a recognized feature. Furthermore, the inflammatory milieu and cellular composition in GBS-affected nerves may differ substantially from the polyneuropathy cases analyzed. Therefore, our cellular source inferences should be considered hypothesis-generating and require validation in GBS nerve tissue or cerebrospinal fluid cellular analyses. While most samples were collected prior to immunomodulatory treatment, a small number of serum samples were obtained after initiation of IVIG (Table S4). Although analyses of the variation in the data suggested moderate effects of IVIG on inflammatory protein profiles in the CSF (but not in the serum), neither IVIG nor PEX confounded the comparative analysis between Control-GBS and COVID-GBS patients (Figure S3). While we accounted for clinical variables such as ICU admission, mechanical ventilation, and timing of sampling, we cannot exclude the possibility that COVID-19 severity, hospitalization, or other unmeasured clinical factors contributed to observed proteomic differences between groups. This represents a potential limitation of our study.

In summary, our findings provide a comprehensive characterization of GBS patients and advance our understanding of its immunopathology. We confirmed elevated IL-8 levels in the CSF of GBS patients and identified LIF, an IL-6 family cytokine, as a novel CSF marker in GBS. Analysis of publicly available single-nucleus transcriptomic data from non-GBS sural nerve biopsies suggested that IL-8 may be produced by myeloid cells within peripheral nerves that also express its receptors, indicating potential autocrine signaling capacity. Similarly, this analysis indicated that LIF receptor components are expressed on endothelial cells and stromal populations, suggesting these as potential cellular targets, though validation in GBS-affected tissue is required. Additionally, we uncovered an inflammatory serum protein signature specific to SARS-CoV-2–associated GBS, indicating potentially distinct pathomechanisms. Together, these results underscore the complex interplay of inflammatory mediators in the pathophysiology of GBS, with distinct mechanisms differentiating COVID-19-related from non-COVID GBS, where NK cell and T cell dysregulation and innate myeloid mechanisms may play a key role in the pathogenesis of COVID-GBS. These findings provide a foundation for future studies exploring therapeutic strategies tailored to infection-dependent inflammatory pathways in GBS.

## Supplementary information

Below is the link to the electronic supplementary material.

Supplementary Material 1

## Data Availability

Data generated during this study has been deposited at Mendeley Data (V1, doi: 10.17632/hd7v7hf3kp.1) and is publicly available as of the date of publication. Source code used to reproduce the results and analyses presented in this manuscript can be found at https://github.com/canulutekin/COVID-GBS. Clinical data will be made available upon reasonable request by the corresponding author.
