# Supplementary material for: Proteomic analysis reveals divergent inflammatory mechanisms of COVID-associated Guillain-Barré syndrome
Source: J Mol Med (Berl). 2026 Jul 4;104(1):93. doi: 10.1007/s00109-026-02698-2 (PMC13331871; doi:10.1007/s00109-026-02698-2)
Supplement: Supplementary file 1 — Supplementary Material 1 [file 109_2026_2698_MOESM1_ESM.docx]

**Supplemental Information**

**Proteomic analysis reveals divergent inflammatory mechanisms in COVID-associated Guillain-Barré syndrome**

Can Ulutekin, Amelie Can, Lenka Súkeníková, Luis Querol, Raul Juntas-Morales, Arnau Llauradó, Anelia Dietmann, Tobias Weiss, Daniela Latorre, Burkhard Becher, Florian Ingelfinger*, Bettina Schreiner*

* These authors contributed equally

**This document includes:**

Clinical characterization

Tables S1 – S5

Figures S1 – S8

**Clinical characterization of COVID-19-related GBS patients and controls**

We evaluated 15 patients with confirmed SARS-CoV2 infection who subsequently developed GBS between 2020 and 2023 (**Tables S1-S5, Figure S1**) treated at University Hospitals Zurich and Bern, Switzerland, and Vall d’Hebron University Hospital, Barcelona, Spain. All COVID-GBS patients had confirmed SARS-CoV2 infection by PCR (**Table S1**). Patients presented with typical respiratory symptoms including cough, shortness of breath, and fever. One patient reported headache, and one patient also had gastrointestinal symptoms with positive C. jejuni serology. Nine patients (60%) were hospitalized due to COVID-19, and three (20%) experienced complications such as ICU stay, acute respiratory distress syndrome (ARDS), and intubation. The average time between COVID-19 and onset of GBS was 25 days (range 10-50 days) (**Table S1**).

COVID-19-related GBS patients mostly displayed a sensorimotor phenotype (67%), with some presenting Miller Fisher Syndrome (MFS)-GBS overlap, ataxic syndrome, or other variants (**Table S1**). Cranial nerve involvement was common (53%), especially facial nerve palsy. Neurological disability ranged from minor symptoms to requiring assisted ventilation, with an average GBS disability score of 3 at nadir (**Table S1**). Nerve conduction studies revealed a predominantly demyelinating GBS type (73%). Five patients (33%) were admitted to ICUs, and four (27%) required mechanical ventilation; one ICU admission was primarily due to complicated COVID-19 (**Table S1**). At last follow-up, recovery was generally favorable with an average GBS disability score of 1 (**Table S1**). No patients died.

Spinal fluid analysis was performed on average 13 days after neurological symptom onset (range 1-28 days) (**Table S2**). All patients showed elevated CSF protein consistent with albuminocytologic dissociation. One patient had SARS-CoV2 RNA detected by viral metagenomics in CSF, but this was not confirmed by PCR (**Table S2**).

For comparison, archived serum and CSF samples from 14 control GBS patients without COVID-19, matched for age and sex, were analyzed (**Table S5**). These patients had similar preceding symptoms and clinical phenotypes, predominantly sensorimotor demyelinating polyneuropathy. Lumbar puncture timing and CSF findings were comparable between groups (**Tables S1-S2**).

Additional control groups included patients with non-inflammatory neuropathies and patients with neurological manifestations unrelated to GBS who underwent lumbar puncture during the pandemic (**Tables S3**). The neuropathy-no-GBS cohort consisted of 18 patients with various forms of non-inflammatory neuropathy, diagnosed based on clinical presentation and nerve conduction studies, with genetic testing performed where applicable. Inclusion criteria required a confirmed diagnosis of one of the neuropathies listed in **Table S3**. Exclusion criteria included a history of GBS, other acute autoimmune disorders, infectious causes of neuropathy, or ongoing immunotherapy at the time of sample collection. All inclusion and exclusion criteria were verified across collaborating centers.

|  | **COVID-GBS (n=15)** | **Control-GBS (n=14) **** | **Neuropathy-no-GBS (n=18)** | **COVID-no-GBS (n=10)** |
| --- | --- | --- | --- | --- |
| **Age** (mean, range) | 56 (44-72) | 58 (21-85) | 59 (21-85) | 51 (29-75) |
| **Sex** (number, %) |  |  |  |  |
| Female | 4 (27%) | 5 (36%) | 6 (33%) | 4 (40%) |
| Male | 11 (73%) | 9 (64%) | 12 (67%) | 6 (60%) |
| **Clinical GBS Type** |  |  |  |  |
| Sensorimotor | 10 (67%) | 11 (79%) |  |  |
| Facial diplegia | 2 (13%) | 0 |  |  |
| MFS-GBS overlap | 1 (7%) | 1 (7%) |  |  |
| Pure sensory | 0 | 1 (7%) |  |  |
| Ataxic | 1 (7%) | 0 |  |  |
| Pharyngocervico-brachial, bilateral nerve palsy | 1 (7%) | 1 (7%) |  |  |
| **GBS symptoms** |  |  |  |  |
| Cranial nerve involvement at onset | 8 (53%) | 5 (36%) |  |  |
| Oculomotor | 2/8 | 1/5 |  |  |
| Facial | 5/8 | 4/5 |  |  |
| Bulbar | 1/8 | 2/5 |  |  |
| Lowest MRC sum score at peak * | 42 (0-60) | 50 (23-60) |  |  |
| Tetraparesis | 9/15 | 9/14 |  |  |
| Paraparesis | 8/15 | 8/13 |  |  |
| Sensory deficits | 14/15 | 12/14 |  |  |
| Pain | 6/14 | 8/14 |  |  |
| Ataxia | 10/15 | 7/13 |  |  |
| Autonomic dysfunction | 1/15 | 2/14 |  |  |
| **GBS disability score** (mean, range) | 3 (1-5) | 3 (1-5) |  |  |
| **Electrophysiology GBS type** (number, %) |  |  |  |  |
| Demyelinating | 11 (73%) | 12 (86%) |  |  |
| Axonal | 2 (13%) | 1 (7%) |  |  |
| Equivocal | 2 (13%) | 1 (7%) |  |  |
| **Treatment** (number, %) |  |  |  |  |
| IVIG | 15/15 (100%) | 12/14 (86%) |  |  |
| Plasma exchange | 6/15 (40%) | 2/14 (14%) |  |  |
| None | 0/15 (0%) | 2/14 (14%) |  |  |
| **ICU admission** (number, %) | 5/15 (33%) *** | 1/14 (7%) |  |  |
| Mechanical ventilation | 4/15 (27%) | 1/14 (7%) |  |  |
| **Recovery** (at last clinical follow-up; mean, range) |  |  |  |  |
| MRC sum score | 54 (0-60) **** | 59 (44-60) |  |  |
| GBS disability score | 1 (0-4) | 1 (0-3) |  |  |
| **Preceding symptoms** (number, %) |  |  |  |  |
| Fever | 5 (33%) | 2 (14%) |  |  |
| Respiratory | 14 (93%) | 6 (43%) ***** |  |  |
| Gastrointestinal | 1 (7%) | 2 (14%) |  |  |
| Headache | 0 (0%) | 2 (14%) |  |  |
| None | 0 (0%) | 5 (36%) |  |  |
| **Days before onset GBS** (mean, range) | 25 (10-50) | 13 (5-28) |  |  |
| **Positive nasopharyngeal PCR for SARS-CoV2** | 15 (100%) |  |  |  |
| **Hospitalized due to COVID-19** |  |  |  |  |
| yes | 9 (60%) |  |  | 5 (50%) |
| no | 6 (40%) |  |  | 5 (50%) |
| **COVID-19 complications** |  |  |  |  |
| yes | 3 (20%) |  |  | 0 (0%) |
| no | 11 (73%) |  |  | 10 (100%) |

**Table S1.** Characteristics of patients used in the study.

MFS = Miller-Fisher syndrome, * MRC sum score (ranging from 60 = normal to 0 = quadriplegic) is the sum of Medical Research Council scores for muscle groups including shoulder abduction, elbow flexion, wrist extension, hip flexion, knee extension, and ankle dorsiflexion of both limbs. The reported score represents the lowest MRC sum score observed during the acute GBS phase. GBS disability score was used to assess GBS severity (range 0–6): 0 = healthy; 1 = minor symptoms but capable of running; 2 = able to walk 10 m without assistance but unable to run; 3 = able to walk 10 m with help; 4 = bedridden or chair-bound; 5 = requiring assisted ventilation for at least part of the day; 6 = dead.^1^ ** Clinical information on GBS type, symptoms, and disability was missing for one patient in the Control-GBS cohort. IVIG = intravenous immunoglobulins. *** One patient’s ICU stay was primarily due to a complicated COVID-19 course. **** Follow-up clinical recovery data were available for 14 COVID-GBS patients and 13 Control-GBS patients. A GBS disability score of 1 corresponds to “minor symptoms but capable of running.” No patient died in either GBS group. Respiratory symptoms included cough and shortness of breath. ***** Includes one patient with respiratory syncytial virus (RSV) infection. SARS-CoV-2 PCR tests were performed between March 2020 and April 2023, all resulting positive. One patient in the COVID-GBS group also had gastrointestinal symptoms and positive C. jejuni serology. COVID-19 complications included intensive care unit (ICU) stay, acute respiratory distress syndrome (ARDS), and intubation. n.a. = not applicable.

|  | **COVID-GBS (n=15)** | **Control-GBS (n=14)** | **Neuropathy-no-GBS (n=18)** | **COVID-no-GBS (n=10)** |
| --- | --- | --- | --- | --- |
| **Time of onset of GBS symptoms to LP** (days, mean, range) | 13 (1-28) | 11 (1-31) |  |  |
| **Cell counts, chemistry** |  |  |  |  |
| Cell count (cells/μL) | 2 (0-6) | 3 (0-24) | 2 (1-3) | 2.5 (0-10) |
| Protein (g/L) | 1.6 (0.5-5.8) | 0.8 (0.2-1.7) | 0.5 (0.2-0.8) | 0.4 (0.3-0.6) |
| Protein elevated (>0.45 g/L) | 15 (100%) | 11 (79%) | 7 (40%) | 3 (30%) |
| **Detection of SARS-CoV2** (in CSF) |  |  |  |  |
| Positive PCR | 0 of 5 tested * |  |  |  |
| **Anti-ganglioside antibody screening** (in serum) |  |  |  |  |
| Positive result (number) | 1/12 | 3/12 | 1/8 |  |

**Table S2.** CSF and routine serum studies of patients used in the study.

* Few reads for SARS-CoV-2 were detected in the CSF of one patient by viral metagenomics analysis; however, this result could not be confirmed by specific PCR.

| **Neuropathy-no-GBS cohort (n=18)** |
| --- |
| Idiopathic axonal or unclassified polyneuropathy (n = 2) |
| Axonal-demyelinating polyneuropathy, diabetes mellitus (n = 3) |
| Axonal-demyelinating polyneuropathy (n = 2) |
| Charcot Marie Tooth neuropathy (n = 11) |
| **COVID-no-GBS (n=10)** |
| Fatigue (n = 3) |
| Cerebrovascular stroke (n = 3) |
| Headache (n = 1) |
| Epileptic seizure (n = 1) |
| Functional disorder (n = 1) |

**Table S3.** Diagnoses of Neuropathy-no-GBS patients and reasons for lumbar puncture of COVID-no-GBS patients.

The time interval between COVID-19 and lumbar puncture (LP) for neurological (non-GBS) manifestations was 58 days on average (range: 1-113 days). The indications for LP and the respective number of patients are detailed in the table. Two COVID-no-GBS patients had oligoclonal bands (OCB) in both serum and CSF, indicating a systemic immune response. All patients reported typical COVID-19 symptoms beforehand, including fever, respiratory symptoms, fatigue, anosmia, headache, and myalgia; one patient also experienced cough and diarrhea. Five patients were hospitalized, none due to severe COVID-19 pneumonia, but rather for neurological diagnostic work-up.

|  | **COVID-GBS (n=15)** | **Control-GBS (n = 14)** |
| --- | --- | --- |
| **IVIG Status** |  |  |
| Untreated | 0 (0%) | 2 (14%) |
| Sampled Before | 9 (60%) | 9 (64%) |
| Sampled After | 6 (40%) | 3 (21%) |
| **PEX Status** |  |  |
| Untreated | 9 (60%) | 12 (86%) |
| Sampled Before | 4 (27%) | 2 (14%) |
| Sampled After | 2 (13%)* | 0 (0%) |
| **Corticosteroid Status** |  |  |
| Untreated | 15 (100%) | 14 (100%) |

**Table S4.** GBS sampling time in relation to various treatments undergone by the patients. * Only one patient had CSF and serum samples collected immediately after 1-2 PEX sessions. In another patient, the serum sample was collected at a time point sufficiently remote from prior PEX such that no relevant treatment-related effects on circulating inflammatory protein markers were expected.

|  | **COVID-GBS (n=15)** | **Control-GBS (n=14)** | **Neuropathy-no-GBS (n=18)** | **COVID-no-GBS (n=10)** |
| --- | --- | --- | --- | --- |
| CSF | 4 of 4 passed QC | 12 of 13 passed QC | 9 of 10 passed QC | 7 of 9 passed QC |
| Serum | 11 of 15 passed QC | 13 of 14 passed QC | 13 of 18 passed QC | 8 of 9 passed QC |

**Table S5.** Sample counts used in the study for each patient group.

Only samples that passed the quality control (QC) were analyzed.

**Figure S1:** Overview of samples from COVID-19 GBS patient and control cohorts, related to Supplementary Table 1.

The flow chart shows the numbers of patients, serum, and cerebrospinal fluid (CSF) samples obtained from cohorts of GBS patients with or without prior COVID-19, as well as control neuropathies. The Zurich cohort included 33 patients: 4 with COVID-GBS, 10 with Control-GBS (occurring before the first reported GBS case in Switzerland in February 2020), 10 with non-inflammatory neuropathies, and 9 with COVID-19 and other neurological manifestations. All except two patients provided paired serum and CSF samples - one COVID-GBS patient had missing CSF, and one COVID-no-GBS patient had missing serum. The Bern cohort included 8 patients: 5 with COVID-GBS (4 serum samples, 1 CSF sample) and 3 with pandemic-era GBS (presumed non-COVID; all three tested negative for SARS-CoV-2 by PCR), with 3 serum and 3 CSF samples collected. The Santa Pau cohort consisted of 8 Neuropathy-no-GBS patients, while the Hebron cohort included 6 COVID-GBS patients, 1 Control-GBS patient, and 1 COVID-no-GBS patient.


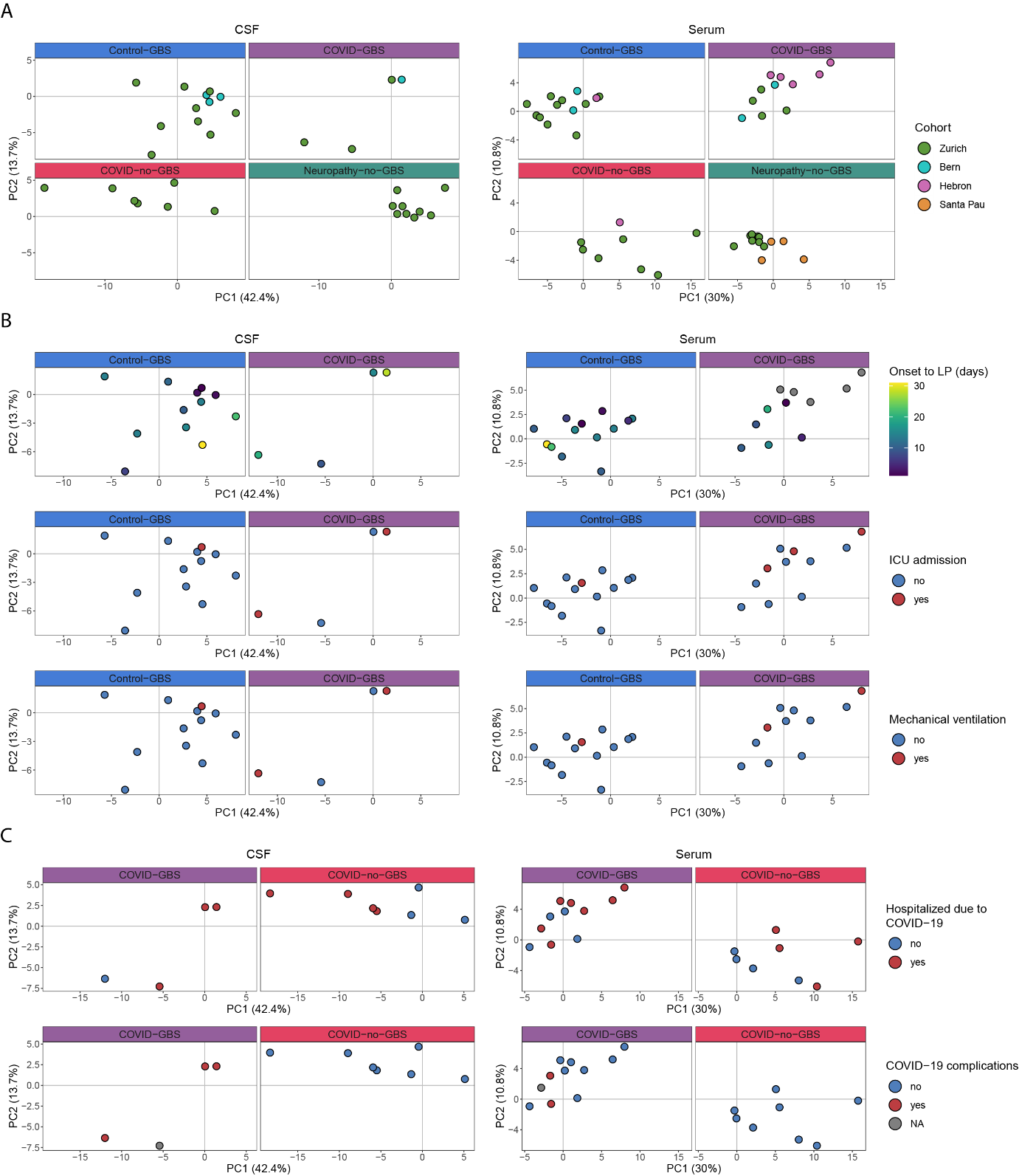


**Figure S2:** PCA analysis of cohort distribution and disease severity.

**(A)** PCAs based on scaled expression of markers, where each dot denotes one patient. Left are CSF samples and right are serum samples. Color code denotes cohort of origin and sub-plot label denotes disease of the patient.

**(B)** PCAs based on scaled expression of markers, where each dot denotes one patient. Left are CSF samples and right are serum samples. Color code denotes various GBS-related clinical parameters and sub-plot label denotes disease of the patient.

**(C)** PCAs based on scaled expression of markers, where each dot denotes one patient. Left are CSF samples and right are serum samples. Color code denotes COVID-19-related clinical parameters and sub-plot label denotes disease of the patient.


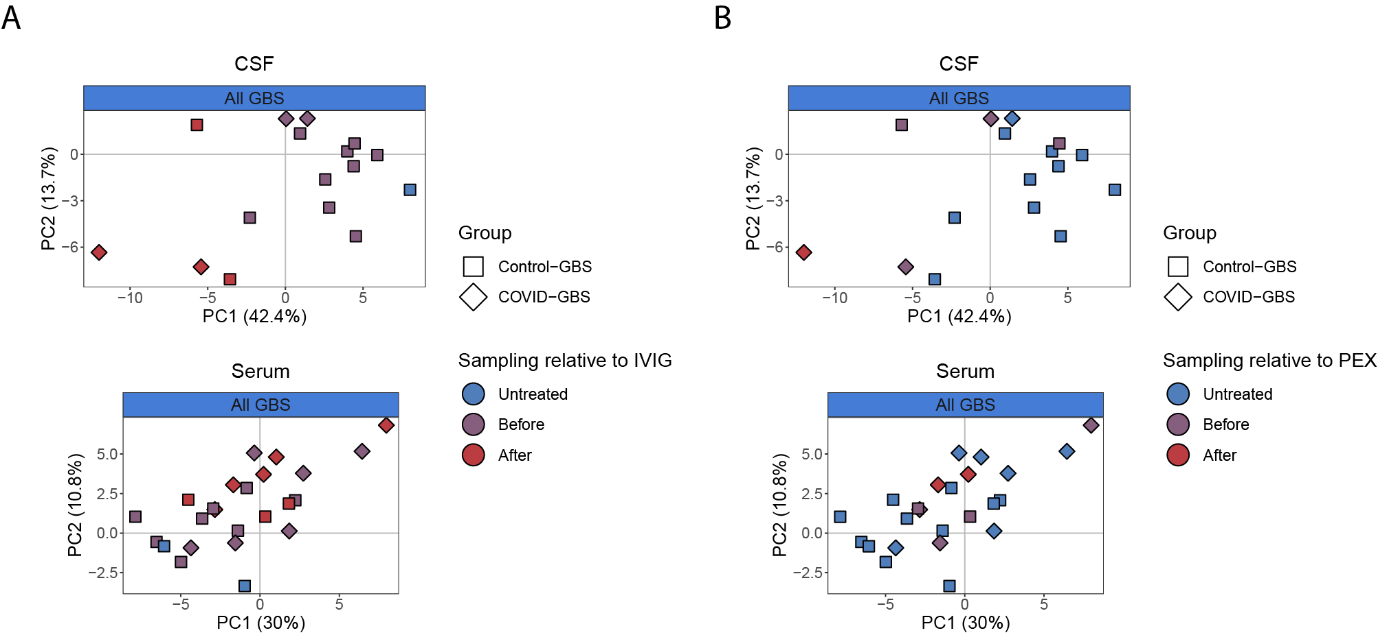


**Figure S3:** PCA analysis of sampling time from treatment.

**(A)** PCAs based on scaled expression of markers, where each dot denotes one patient. Top panel is the CSF samples and bottom panel is the serum samples. Color code denotes cohort of sampling in relation to intravenous immunoglobulin (IVIG) treatment. Dot shape denotes disease group classification.

**(B)** PCAs based on scaled expression of markers, where each dot denotes one patient. Top panel is the CSF samples and bottom panel is the serum samples. Color code denotes cohort of sampling in relation to plasma exchange (PEX) treatment. Dot shape denotes disease group classification.


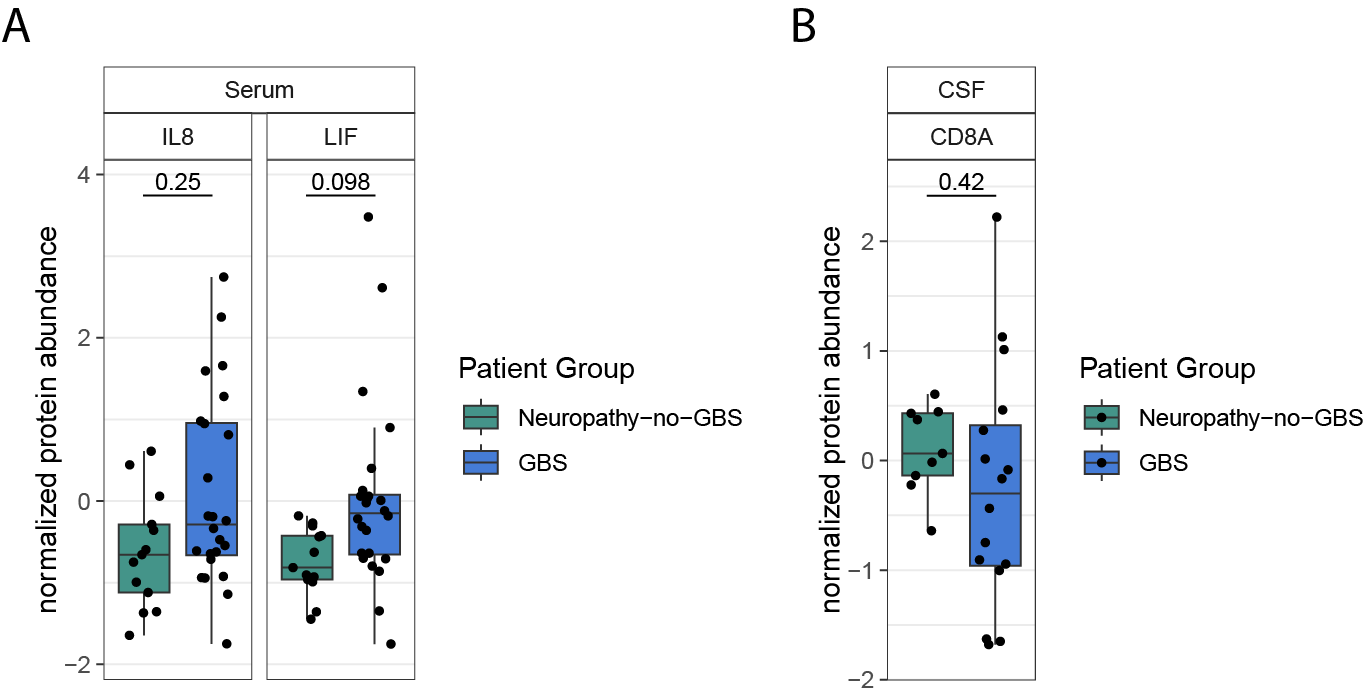


**Figure S4:** Cross-compartment validation of GBS-associated protein markers in serum and CSF

**(A)** Boxplots comparing serum scaled expression of markers identified in the CSF as distinguishing between GBS and Neuropathy-no-GBS patients. Unpaired Wilcoxon rank-sum test and Benjamini-Hochberg correction were applied.

**(B)** Boxplots comparing CSF scaled expression of markers identified in the serum as distinguishing between GBS and Neuropathy-no-GBS patients. Unpaired Wilcoxon rank-sum test and Benjamini-Hochberg correction were applied.


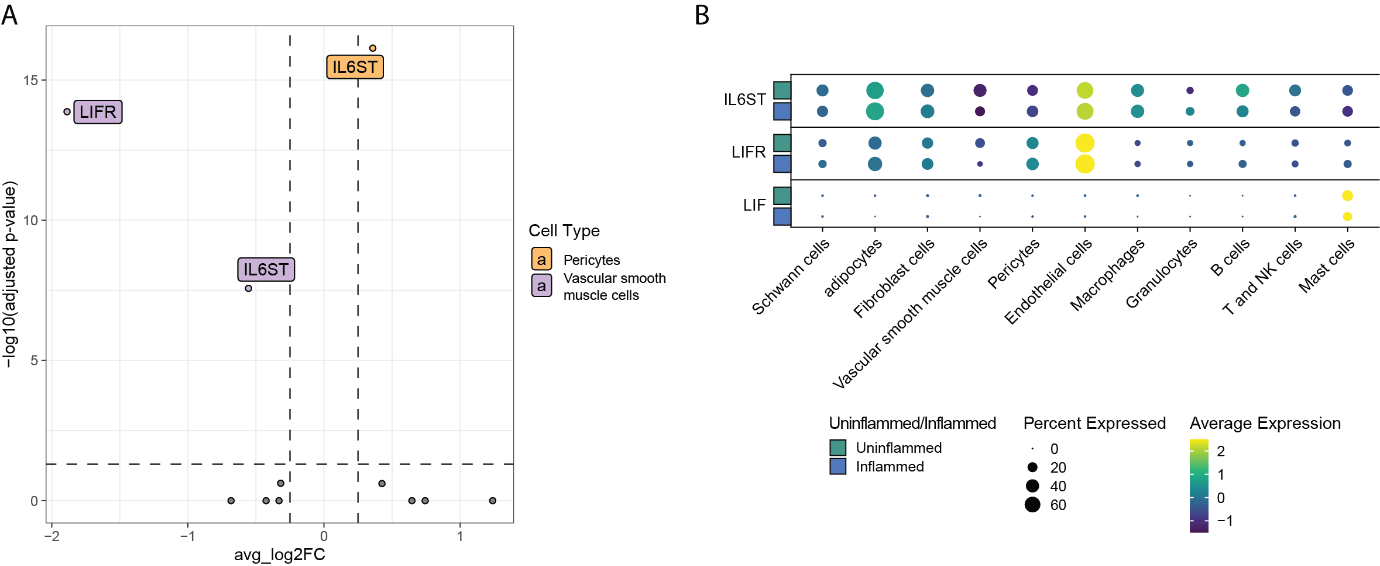


**Figure S5:** Differential gene expression in sural nerve biopsies from inflammatory versus uninflammatory neuropathies.

**(A)** Volcano plot showing differential gene expressions between uninflammatory and inflammatory neuropathies across identified clusters. Genes were pre-filtered to include only those expressed in at least 10% of cells in either condition. Statistical significance was assessed using the unpaired Mann-Whitney-Wilcoxon test, with multiple testing correction applied via the Benjamini-Hochberg method. Genes with a log₂ fold change ≥ 0.25 and an adjusted p-value ≤ 0.05 were considered significant.

**(B)** Dot plot illustrating gene expression of LIF along with its receptors LIFR and IL6ST across major identified clusters and comparing uninflammatory and inflammatory neuropathies. Dot size indicates the proportion of nuclei expressing each gene within a cluster; color intensity reflects the average expression level.


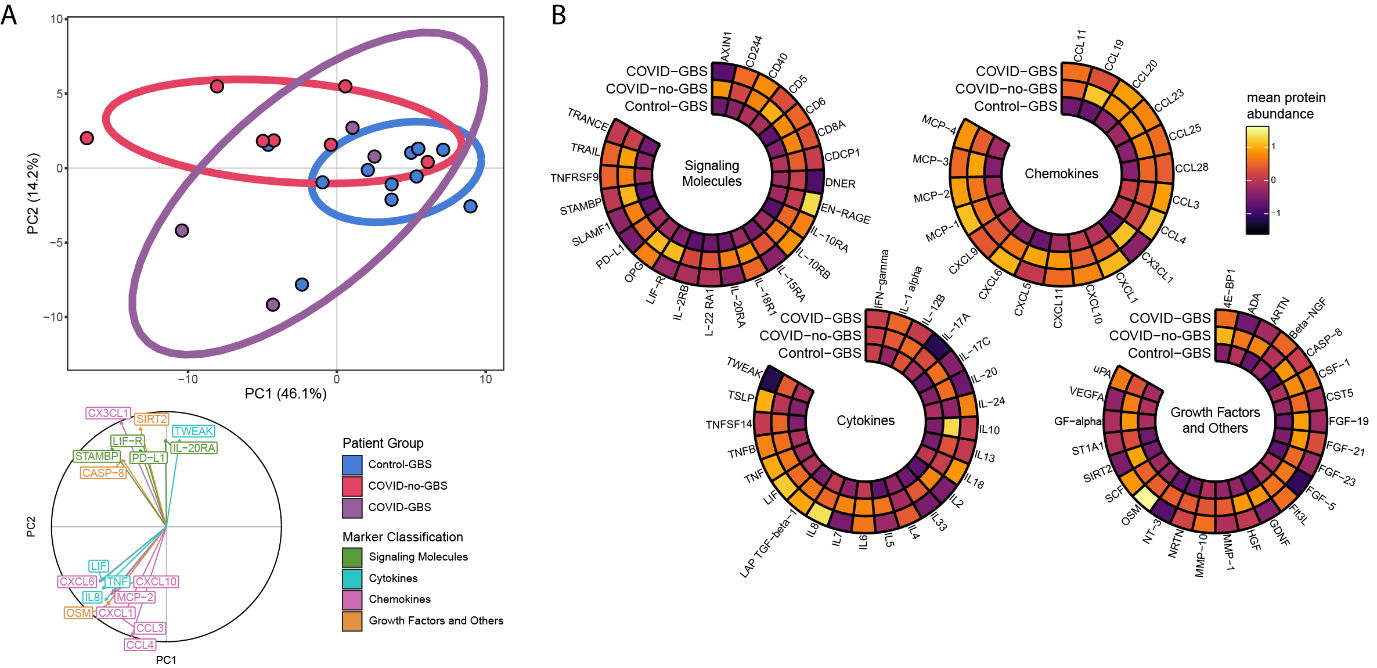


**Figure S6:** Preliminary, hypothesis-generating CSF analysis comparing COVID-GBS patients to Control-GBS and COVID-no-GBS patients.

**(A-B)** CSF samples from 4 COVID-GBS patients were compared descriptively to those from 7 COVID-no-GBS patients and 12 Control-GBS patients.

**(A)** PCA based on scaled expression of markers, where each dot denotes one patient. Vectors of markers in the circle denote top contributors to PC1 and PC2. PCA results are shown for exploratory visualization only.

**(B)** Heatmaps illustrating mean scaled marker expressions comparing COVID-GBS, COVID-no-GBS and Control-GBS patients. Markers are assigned to mutually exclusive categories for ease of representation. No statistical testing was performed.


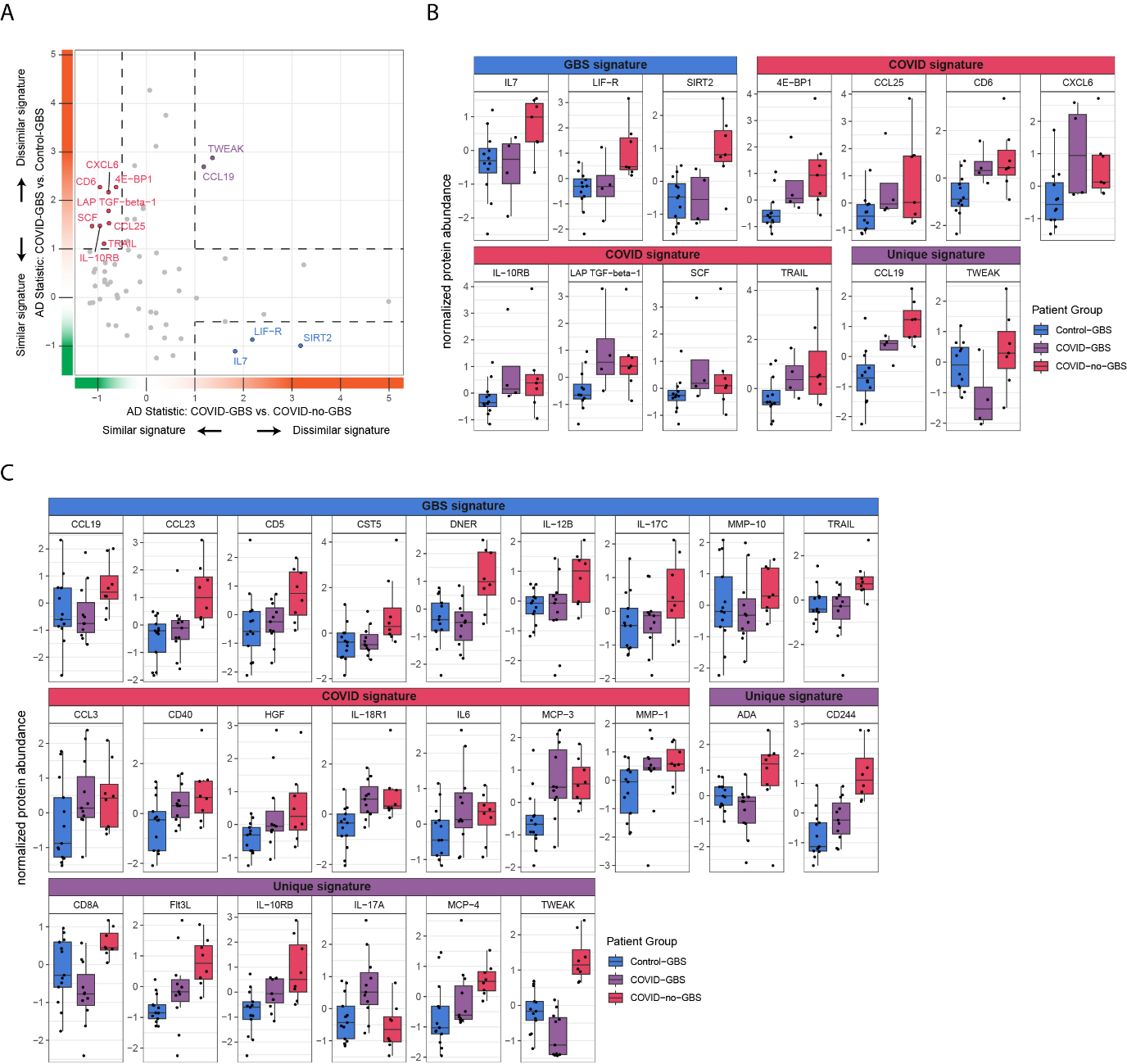


**Figure S7:** Serum protein profiles reveal shared and distinct immune signatures in COVID-GBS compared to control groups.

(**A-B**) Preliminary, hypothesis-generating analysis of CSF

**(A)** CSF samples of 4 COVID-GBS, 7 COVID-no-GBS and 12 Control-GBS patients. Scatter plot showing Anderson-Darling (AD) statistics comparing protein expression distributions. X-axis represents the AD statistic for COVID-GBS versus Control-GBS; Y-axis represents the AD statistic for COVID-GBS versus COVID-no-GBS. Lower (more negative) values indicate greater similarity in distribution between groups, while higher (more positive) values indicate distinct distributions. Cut-offs: -0.5 for similarity, 1.0 for dissimilarity.

**(B)** CSF samples of 4 COVID-GBS, 7 COVID-no-GBS and 12 Control-GBS patients. Boxplots comparing scaled expression levels among COVID-GBS, Control-GBS, and COVID-no-GBS groups.

**(C)** Serum samples of 11 COVID-GBS, 8 COVID-no-GBS and 13 Control-GBS patients. Boxplots comparing scaled expression levels among COVID-GBS, Control-GBS, and COVID-no-GBS groups.


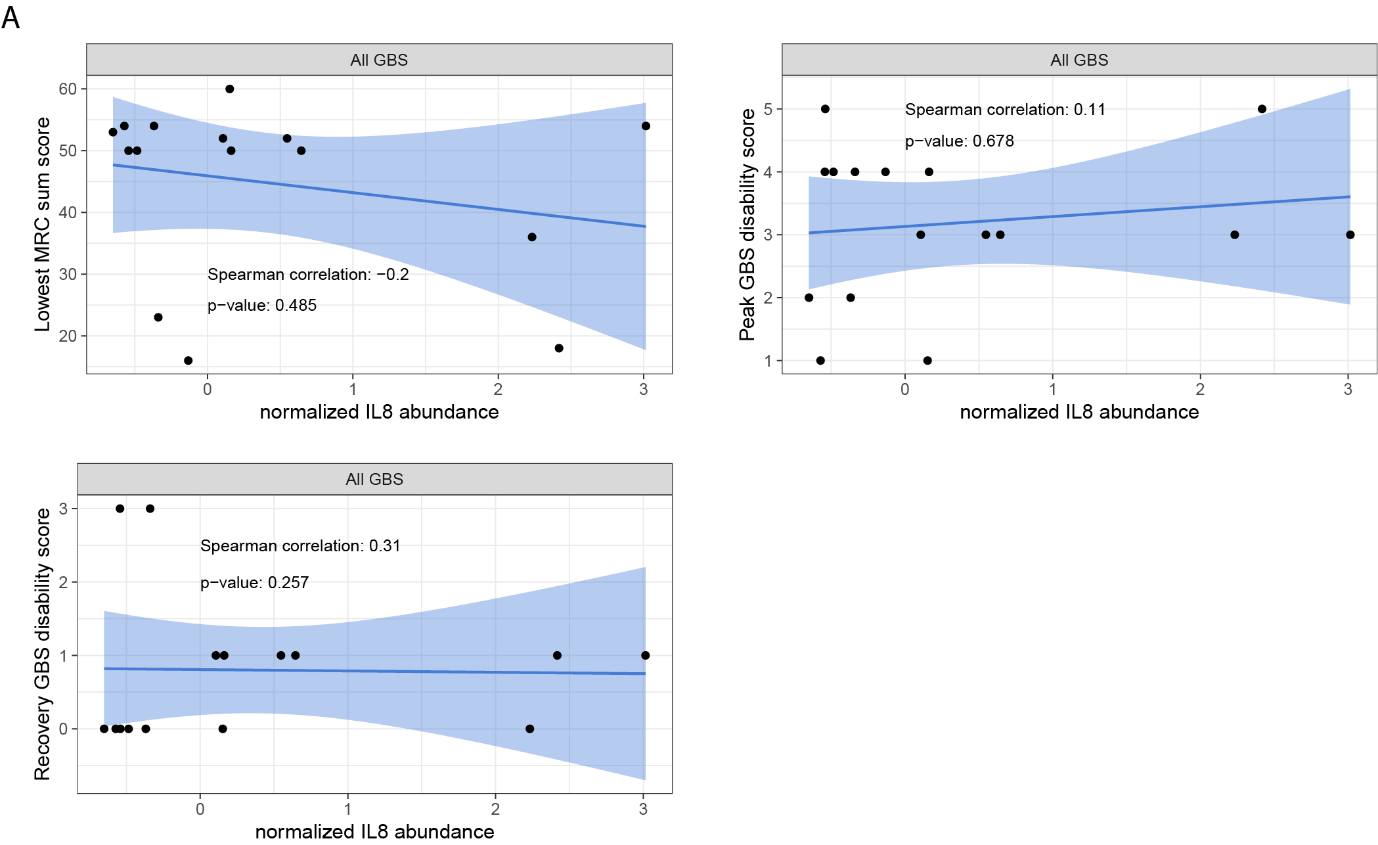


**Figure S8:** Relationships between CSF IL-8 Levels and Clinical Outcome Measures in GBS

**(A)** Scatter plots displaying scaled CSF IL-8 expression (x-axis) in relation to three clinical outcome measures: lowest MRC sum score (top-left panels), peak GBS disability score (top-right panels) and recovery GBS disability score (bottom panels), shown for GBS (Control-GBS, and COVID-GBS combined). Each dot represents an individual patient. Trendlines depict the direction of the association, and Spearman correlation coefficients with corresponding p-values are provided within each panel.

**Supplementary References**

1. Hughes RAC, Newsom-Davis JM, Perkin GD, Pierce JM. CONTROLLED TRIAL OF PREDNISOLONE IN ACUTE POLYNEUROPATHY. *The Lancet*. 1978;312(8093):750-753. doi:10.1016/S0140-6736(78)92644-2
